# Supplementary material for: Human histone fragments display antibacterial properties against Pseudomonas aeruginosa
Source: Front Immunol. 2026 Aug 12;17:1879460. doi: 10.3389/fimmu.2026.1879460 (PMC13506288; doi:10.3389/fimmu.2026.1879460)
Supplement: Supplementary file 1 [file DataSheet1.pdf]

Supplementary Tables

Supplementary Tables.

Table 1. Semi-quantitative analysis of RT-PCR. Relative fold change of *SecA* and *SecY* expression levels after the normalization of adjusted band volume values to housekeeping gene (*rpsl30*) among H1.2-treated and untreated samples at different RNA concentrations.

Table 1. Semi-quantitative analysis of RT-PCR

RNA concentration (ng/µl)	SecA	SecY
10	0,94934	1,00165
5	1,00386	0,95955
2.5	0,96754	0,88363
1.25	0,99902	0,8732
0.625	1,3529	1,07392

Table 2 and Table 3. Detailed analysis of proteomics data. MaxQuant analysis using UniProt *P. aeruginosa* shows some up-regulated **(Table 2)** and down-regulated **(Table 3)** proteins after treatment of bacteria with H1.2 peptide. The tables show the differentially expressed proteins, including their UniProt IDs, gene names, biological function, and fold changes (FC).

Table 2: Up-regulated Proteins

Protein ID	Protein name	Gene	Biological function	FC
O54439	Acyl carrier protein 1	*acpP1*	fatty acid biosynthesis	4,946
Q9HTX6	Glycine cleavage system H protein 2	*gcvH2*	glycine decarboxylation	3,153
Q9HTQ4	Cytochrome c5	*cycB*	electron transport	3,042
P11724	Ornithine carbamoyltransferase, anabolic	*argF*	amino-acid biosynthesis	2,536
Q9HW32	Insulin-cleaving metalloproteinase outer membrane protein	*icmP*	metal ion binding (mol)	2,350

E1JGJ8	Peptide chain release factor 2	*prfB*	protein biosynthesis	2,266
Q9X2T1	Thioredoxin	*trxA*	electron transport	2,092
Q9HV46	Transcription elongation factor GreA	*greA*	transcription	1,906
Q9HWD5	Large ribosomal subunit protein uL3	*rplC*	translation	1,895
P00282	Azurin	*azu*	electron transport	1,772
Q9I0A0	Translation initiation factor IF-3	*infC*	protein biosynthesis	1,629
Q9HWF4	Large ribosomal subunit protein uL15	*rplO*	translation	1,611
Q9HUN2	Large ribosomal subunit protein bL9	*rplI*	translation	1,444
O82853	Ribosome-recycling factor	*frr*	protein biosynthesis	1,423
Q9I2U9	Peptidyl-prolyl cis-trans isomerase	*ppiB*	protein folding	1,412
P95459	Major cold shock protein CspA	*cspA*	stress response	1,410
P13982	Carbamate kinase	*arcC*	arginine metabolism	1,402
O82851	Elongation factor Ts	*tsf*	protein biosynthesis	1,393
P57668	Thiol peroxidase	*tpx*	cellular response	1,387
Q9HZC7	DUF1329 domain-containing protein		unknown	1,381
Q9HUD0	Large ribosomal subunit protein bL31	*rpmE*	translation	1,380
Q9HV60	BON domain-containing protein		response to osmotic stress	1,360
P29363	Threonine synthase	*thrC*	amino-acid biosynthesis	1,353
Q9HWE4	Small ribosomal subunit protein uS17	*rpsQ*	translation	1,341

Q9HU11	Peptidoglycan-binding protein LysM		response to potassium ion	1,326
Q9HWC7	Large ribosomal subunit protein uL10	*rplJ*	translation	1,325
Q9HVI7	Serine hydroxymethyltransferase 3	*glyA3*	amino-acid biosynthesis	1,309
Q9I3B2	3-oxoacyl-[acyl-carrier-protein] synthase 1	*fabB*	fatty acid biosynthesis	1,295
Q9HUM2	Protein HflK	*hflK*	proteolysis	1,280
Q9HXU9	DUF4398 domain-containing protein		unknown	1,278
Q9HTR6	Nitrogen regulatory protein P-II 2	*glnK*	cellular response to antibiotic	1,237
Q9HZP7	Electron transfer flavoprotein subunit alpha	*etfA*	electron transport	1,219
Q9I5E3	Citrate synthase	*prpC*	carbohydrate metabolic process	1,209

Table 3: Down-regulated Proteins

Protein IDs	Protein name	Gene	Biological function	FC
Q9I5A7	OmpA-like domain-containing protein		monoatomic ion transport	0,800
Q9HVV7	Lipopolysaccharide export system protein LptH	*lptH*	lipopolysaccharide tranposrt	0,798
Q9HXV4	Adenylate kinase	*adk*	nucleotide biosynthesis	0,797
Q9HT20	ATP synthase subunit beta	*atpD*	atp synthesis	0,795
Q9I671	glutaryl-CoA dehydrogenase (ETF)	*gcdH*	biosynthetic process	0,794
P09591	Elongation factor Tu	*tufA*	translation elongation	0,789
Q9HVY4	Ubiquinol-cytochrome c reductase iron-sulfur subunit		oxidoreductase activity	0,787
Q9I685	Adenosylhomocysteinase	*ahcY*	metabolic process	0,784

Q9I2V5	Aconitate hydratase B	*acnB*	tricarboxylic acid cycle	0,781
P53641	Superoxide dismutase [Fe]	*sodB*	superoxide dismutase activity	0,777
Q9HVA2	Ketol-acid reductoisomerase (NADP(+))	*ilvC*	biosynthetic process	0,776
Q9HVT7	Aspartyl/glutamyl-tRNA(Asn/Gln) amidotransferase subunit B	*gatB*	translation	0,772
Q9I0L4	Isocitrate dehydrogenase [NADP]	*idh*	oxidoreductase (mol)	0,771
Q9HWD1	Small ribosomal subunit protein uS7	*rpsG*	translation	0,765
Q9HTN8	Large ribosomal subunit protein bL28	*rpmB*	translation	0,764
Q9HVY3	Small ribosomal subunit protein uS9	*rpsI*	translation	0,764
Q9HYK7	ferredoxin--NADP(+) reductase	*fpr*	catabolic process	0,763
O30508	Succinylornithine transaminase/acetylornithine aminotransferase	*aruC*	biosynthetic process	0,758
Q9HTZ7	Phosphoenolpyruvate carboxykinase	*pckA*	gluconeogenesis	0,758
Q9HTL9	RidA family protein		catabolic process	0,756
G3XD11	PhoP/Q and low Mg2+ inducible outer membrane protein H1	*oprH*	lipopolysaccharide binding	0,752
Q9I4I1	Ribonucleoside-diphosphate reductase	*nrdA*	deoxyribonucleotide synthesis	0,751
Q9HXM5	Inosine-5'-monophosphate dehydrogenase	*guaB*	biosynthetic process	0,750
Q9HTN2	Acetylglutamate kinase	*argB*	biosynthetic process	0,749
Q9HWC9	DNA-directed RNA polymerase subunit beta'	*rpoC*	dna-templated transcription	0,749
Q9HT18	ATP synthase subunit alpha	*atpA*	atp synthesis	0,748
Q9I3G1	Cytochrome c oxidase, cbb3-type, CcoO subunit	*ccoO2*	cytochrome-c oxidase activity	0,748
Q9I4S1	Glycine zipper 2TM domain-containing protein			0,746
Q51487	Outer membrane protein OprM	*oprM*	response to antibiotic/ transmembrane transport	0,743
Q9HUM5	ATP phosphoribosyltransferase regulatory subunit	*hisZ*	biosynthetic process	0,743

Q9HV42	Protein GrpE	*grpE*	protein folding	0,741
Q9I0L5	Isocitrate dehydrogenase [NADP]	*icd*	oxidoreductase (mol)	0,741
Q9HWE7	Large ribosomal subunit protein uL5	*rplE*	translation	0,740
Q9I2A6	3-hydroxybutyrate dehydrogenase	*bdhA*	oxidoreductase (mol)	0,739
Q9I420	Carboxypeptidase regulatory-like domain-containing protein			0,737
P08308	Ornithine carbamoyltransferase	*arcB*	biosynthetic process	0,732
Q9HZM8	Ribonuclease E	*rne*	trna processing	0,732
Q9HUM9	Small ribosomal subunit protein bS6	*rpsF*	translation	0,731
G3XD30	Type IV pilus inner membrane component PilN	*pilN*	type iv pilus assembly	0,729
Q51567	Succinate--CoA ligase	*sucD*	biosynthetic process	0,728
Q9I5H0	Folate-binding protein		iron-sulfur cluster assembly	0,727
P40947	Single-stranded DNA-binding protein	*ssb*	dna binding	0,723
Q9HVC5	Ribose-phosphate pyrophosphokinase	*prs*	biosynthetic process	0,723
Q9I6Z3	Alkyl hydroperoxide reductase C	*ahpC*	cellular response to stress	0,720
Q9HWE0	Large ribosomal subunit protein uL22	*rplV*	translation	0,718
Q59636	Nucleoside diphosphate kinase	*ndk*	biosynthetic process	0,717
Q9HVL6	Large ribosomal subunit protein bL21	*rplU*	translation	0,717
Q9HVN5	Chaperone protein ClpB	*clpB*	protein refolding	0,716
O52762	Catalase	*katA*	catabolic process	0,714
P37798	Biotin carboxylase	*accC*	biosynthetic process	0,713
Q9I5Q3	Tyrosine--tRNA ligase 2	*tyrS2*	protein biosynthesis	0,712
Q9ZN70	Exopolyphosphatase	*ppx*	biosynthetic process	0,712
O52759	Small ribosomal subunit protein uS4	*rpsD*	translation	0,709

Q9I687	Methylenetetrahydrofolate reductase	*metF*	amino-acid biosynthesis	0,703
Q9HWW1	Outer membrane protein OprG	*oprG*	protein transport	0,703
Q59637	Pyruvate dehydrogenase E1 component	*aceE*	pyruvate dehydrogenase activity	0,700
Q9HWD8	Large ribosomal subunit protein uL2	*rplB*	cytoplasmic translation	0,699
Q9HWD6	Large ribosomal subunit protein uL4	*rplD*	translation	0,696
O50273	Sulfate adenylyltransferase subunit 2	*cysD*	biosynthetic process	0,694
Q9HXP3	Formate-dependent phosphoribosylglycinamide formyltransferase	*purT*	purine biosynthesis	0,692
Q9I5Y4	Phosphoglycerate kinase	*pgk*	glycolysis	0,687
Q9HTP2	Probable aldehyde dehydrogenase		oxidoreductase activity	0,686
P13981	Arginine deiminase	*arcA*	catabolic process	0,685
Q9I0K4	Isocitrate lyase		biofilm formation	0,679
Q9HWD9	Small ribosomal subunit protein uS19	*rpsS*	translation	0,675
Q9HVD1	Lipid A deacylase PagL	*pagL*	metabolic process	0,671
Q9I3D1	Dihydrolipoyl dehydrogenase	*lpdG*	oxidoreductase (mol)	0,670
Q9HVY2	Large ribosomal subunit protein uL13	*rplM*	translation	0,669
Q9HVX1	Probable periplasmic serine endoprotease DegP-like	*algW*	proteolysis	0,668
Q9HWX4	3,4-dihydroxy-2-butanone 4-phosphate synthase	*ribB*	riboflavin biosynthesis	0,665
Q9HWF7	Small ribosomal subunit protein uS13	*rpsM*	translation	0,664
Q9HWD4	Small ribosomal subunit protein uS10	*rpsJ*	translation	0,663
Q9I765	oligopeptidase A	*prlC*	metabolic process	0,663
Q9HTD9	alcohol dehydrogenase	*adhA*	zinc ion binding	0,662
Q9I3G2	Cbb3-type cytochrome c oxidase subunit	*ccoP2*	electron transport	0,660

P38098	Carbamoyl phosphate synthase small chain	*carA*	biosynthetic process	0,656
Q9X6W6	Bifunctional protein PyrR	*pyrR*	transcription	0,656
Q59643	Delta-aminolevulinic acid dehydratase	*hemB*	biosynthetic process	0,653
G3XD89	Copper transport outer membrane porin OprC	*oprC*	transmembrane transport	0,652
Q9I3C5	Chaperone protein HtpG	*htpG*	stress response	0,650
Q9I5Z0	S-adenosylmethionine synthase	*metK*	metabolism	0,649
Q9HU65	Glutamine synthetase	*glnA*	biosynthetic process	0,648
Q9I574	Membrane-bound lysozyme inhibitor of C-type lysozyme	*mliC*		0,648
Q9I4W3	4-hydroxy-tetrahydrodipicolinate synthase	*dapA*	amino-acid biosynthesis	0,646
Q9HVS6	DUF541 domain-containing protein		dna damage response	0,645
Q9HXU0	Lysine--tRNA ligase	*lysS*	protein biosynthesis	0,645
Q9HVW4	Toluene tolerance protein			0,644
Q9HTD0	Biotin carboxylase		biosynthetic process	0,643
Q9I5F6	Bifunctional protein PutA	*putA*	metabolism	0,643
Q9I5U9	PrkA AAA domain-containing protein		protein kinase activity (mol)	0,643
Q9HUA7	Probable binding protein component of ABC transporter			0,637
Q9I0X8	DUF2185 domain-containing protein		unknown	0,637
Q9HU73	Fructose-1,6-bisphosphatase class 1	*fbp*	metabolic process	0,629
Q9HTQ1	RidA family protein		no information	0,628
Q9HZJ5	DNA topoisomerase 1	*topA*	dna-binding (mol)	0,626
O50274	Bifunctional enzyme CysN/CysC	*cysNC*	metabolic process	0,625
Q9HWE1	Small ribosomal subunit protein uS3	*rpsC*	translation	0,624
Q9HYY3	Periplasmic tail-specific protease	*prc*	proteolysis	0,622

Q9HTD1	Probable transcarboxylase subunit		ion transport	0,621
Q9I0M4	Outer-membrane lipoprotein carrier protein	*lolA*	protein transport	0,620
Q9I1R3	Probable binding protein component of ABC transporter		ion transport (mol)	0,618
Q9I405	Amino acid ABC transporter ATP binding protein		transport	0,617
Q9HZ55	Ribosomal large subunit pseudouridine synthase B	*rluB*	translation	0,616
Q9HZ48	Probable binding protein component of ABC sugar transporter		protein transport	0,614
Q9I3F5	Aconitate hydratase A	*acnA*	tricarboxylic acid cycle-electron transport	0,612
Q9I4I2	Ribonucleoside-diphosphate reductase subunit beta	*nrdB*	deoxyribonucleotide synthesis	0,612
Q9HVY6	Probable cytochrome c1		metal ion binding	0,607
Q9I3D5	Succinate dehydrogenase flavoprotein subunit	*sdhA*	electron transport	0,606
Q9I0J5	NADH-quinone oxidoreductase subunit H	*nuoH*	aerobic respiration	0,604
Q9I0H4	Flavohemoprotein	*hmp*	cellular response to stress	0,602
G3XD51	Type IV pilus inner membrane component PilO	*pilO*	type iv pilus assembly	0,601
Q9HX12	MaoC-like domain-containing protein			0,601
Q9HYU8	Probable HIT family protein		metabolic process	0,601
Q9HUC0	Ubiquinone/menaquinone biosynthesis C-methyltransferase UbiE	*ubiE*	biosynthetic process	0,600
Q9HXY5	Skp-like protein		protein folding	0,600
Q9HYC7	Methionine--tRNA ligase	*metG*	protein biosynthesis	0,600
Q03456	Ferric uptake regulation protein	*fur*	dna binding	0,597
Q9HYQ8	ADP-L-glycero-D-manno-heptose-6-epimerase	*hldD*	biosynthetic process	0,597
Q9HY13	Xylulose 5-phosphate/Fructose 6-phosphate phosphoketolase N-terminal domain-containing protein		metabolic process	0,597
Q9HWV5	Probable aldehyde dehydrogenase		phenylacetaldehyde dehydrogenase activity (mol)	0,595

Q9I3D3	oxoglutarate dehydrogenase (succinyl-transferring)	*sucA*	tricarboxylic acid cycle-electron transport	0,594
Q9HV67	Glucose-6-phosphate isomerase	*pgi*	metabolic process	0,593
Q9I4K8	Probable oxidoreductase		oxidoreductase (mol)	0,593
Q9HZK4	Glyceraldehyde-3-phosphate dehydrogenase		metabolic process	0,593
P27726	Glyceraldehyde-3-phosphate dehydrogenase	*gap*	metabolic process	0,590
Q9I5Y1	Fructose-bisphosphate aldolase	*fba*	glycolysis	0,589
Q9HTQ6	Xanthine phosphoribosyltransferase	*xpt*	metabolic process	0,586
Q9HVM4	Isoleucine--tRNA ligase	*ileS*	isoleucyl-trnaaminoacylation	0,586
Q9I0A2	Large ribosomal subunit protein bL20	*rplT*	translation	0,585
P43335	Pterin-4-alpha-carbinolamine dehydratase	*phhB*	biosynthetic process	0,583
Q9HVA1	Acetolactate synthase small subunit	*ilvH*	biosynthetic process	0,580
P43336	Aromatic-amino-acid aminotransferase	*phhC*	catabolic process	0,579
Q9I6J6	Probable periplasmic polyamine binding protein		transport	0,579
Q9I047	Transaldolase	*tal*	metabolic process	0,579
Q9I636	Malate synthase G	*glcB*	catabolic process	0,578
Q9HZ98	Heat-shock protein IbpA	*ibpA*	stress response	0,576
Q9HVL9	Glutamate 5-kinase	*proB*	biosynthetic process	0,574
Q9I4W5	thioredoxin-dependent peroxiredoxin	*bcp*	cellular response to oxidative stress	0,574
Q9HUG9	Bifunctional protein HldE	*hldE*	biosynthetic process	0,573
Q9HV96	Aminoglycoside phosphotransferase domain-containing protein			0,571
P42257	Protein PilJ	*pilJ*	chemotaxis	0,570
Q9HTQ0	D-amino acid dehydrogenase 1	*dadA1*	catabolic process	0,570
Q9HXI4	Nus factor SuhB	*suhB*	metabolic process	0,570

Q9I0K0	NADH-quinone oxidoreductase subunit B	*nuoB*	aerobic respiration	0,570
Q9HXR8	Probable FMN oxidoreductase		oxidoreductase (mol)	0,569
G3XCV0	Transcriptional regulator FleQ	*fleQ*	dna-templated transcription	0,568
Q51551	Dihydroorotase-like protein	*pyrC'*	biosynthetic process	0,567
Q9HVZ9	UDP-N-acetylmuramoylalanine--D-glutamate ligase	*murD*	cell wall organitation	0,567
Q9I5I4	Probable enoyl-CoA hydratase/isomerase		catabolism	0,567
Q9HUK1	DNA topoisomerase 4 subunit A	*parC*	chromosome segregation	0,566
Q9I352	Bacteriohemerythrin		transport	0,566
Q9I1N4	PslE	*pslE*	biofilm formation	0,564
Q9I5E2	2-methylisocitrate lyase	*prpB*	catabolic process	0,564
Q9HWG0	UvrABC system protein A	*uvrA*	dna damage	0,564
Q9I137	Glycine dehydrogenase (decarboxylating) 1	*gcvP1*	oxidoreductase (mol)	0,562
Q9HZK6	Na(+)-translocating NADH-quinone reductase subunit A	*nqrA*	ion transport	0,561
Q9I669	HotDog ACOT-type domain-containing protein		metabolic process	0,560
Q9HXY7	3-hydroxyacyl-[acyl-carrier-protein] dehydratase FabZ	*fabZ*	lipid metabolism	0,560
Q9HZ66	Phosphoserine aminotransferase	*serC*	amino-acid biosynthesis	0,560
Q9HV59	Polyribonucleotide nucleotidyltransferase	*pnp*	catabolic process	0,559
Q9HXN2	Phosphoribosylformylglycinamidine synthase	*purL*	purine biosynthesis	0,557
Q9HU31	Amino acid ABC transporter			0,556
Q9HX91	Uncharacterized protein PA3922		protein biosynthesis	0,556
Q9I4R0	Lipoprotein			0,555
Q9I6H5	D-3-phosphoglycerate dehydrogenase	*serA*	metabolism	0,555
Q9I6M5	Glutarate-semialdehyde dehydrogenase	*davD*	catabolic process	0,555

Q9HXZ5	Enolase	*eno*	glycolysis	0,555
Q9I502	Proline--tRNA ligase	*proS*	protein biosynthesis	0,554
Q9HZA7	Acetyl-coenzyme A carboxylase carboxyl transferase subunit beta	*accD*	fatty acid biosynthesis	0,554
Q9HUM3	Protein HflC	*hflC*	proteolysis	0,553
Q9I0M6	Serine--tRNA ligase	*serS*	protein biosynthesis	0,553
Q9HZE0	NAD-specific glutamate dehydrogenase	*gdhB*	oxidoreductase (mol)	0,553
Q9I0K9	Adenylosuccinate lyase	*purB*	biosynthetic process	0,553
P50599	Tol-Pal system protein TolR	*tolR*	cell division/ protein transport	0,552
Q9HVT8	Glutamyl-tRNA(Gln) amidotransferase subunit A	*gatA*	translation	0,552
Q9I2W9	Phosphoenolpyruvate synthase	*ppsA*	gluconeogenesis	0,552
Q9I6M4	5-aminovalerate aminotransferase DavT	*davT*	catabolic process	0,552
Q9HXH0	Valine--tRNA ligase	*valS*	protein biosynthesis	0,552
G3XDB0	Catabolite repression control protein	*crc*	quorum sensing	0,551
Q9HU22	Glucose-1-phosphate thymidylyltransferase	*rmlA*	biosynthetic process	0,550
P08280	Protein RecA	*recA*	dna binding	0,549
P23189	Glutathione reductase	*gor*	cell redox homeostasis	0,549
P29365	Homoserine dehydrogenase	*hom*	biosynthetic process	0,549
Q9I672	CoA transferase		transferase (mol)	0,549
Q9I099	Threonine--tRNA ligase	*thrS*	protein biosynthesis	0,549
P0C2B2	Thiol:disulfide interchange protein DsbA	*dsbA*	oxidoreductase activity	0,548
P55222	cAMP-activated global transcriptional regulator Vfr	*vfr*	dna binding	0,545
Q9I5E4	aconitate hydratase		metabolic process	0,545
Q9I1N5	PslD	*pslD*	biofilm formation	0,544

Q9HWR2	Aminoglycoside 3'-phosphotransferase	*aph*	antibiotic resistance	0,544
Q9I0J9	NADH-quinone oxidoreductase subunit C/D	*nuoC*	aerobic respiration	0,544
P55218	O-succinylhomoserine sulfhydrylase	*metZ*	biosynthetic process	0,543
Q9I2P8	Fe/S biogenesis protein NfuA	*nfuA*	iron binding (mol)	0,543
Q9I6B4	Thiazole synthase	*thiG*	biosynthesis	0,543
Q9I0J7	NADH-quinone oxidoreductase subunit F	*nuoF*	aerobic respiration	0,542
Q9HVX6	Tryptophan--tRNA ligase	*trpS*	tryptophanyl-trna aminoacylation	0,541
Q9I296	Isovaleryl-CoA dehydrogenase, mitochondrial	*liuA*	catabolic process	0,541
Q9I649	Protein phosphatase 2C domain-containing protein			0,540
O50174	N-succinylglutamate 5-semialdehyde dehydrogenase	*astD*	catabolic process	0,539
Q14T74	proton-translocating NAD(P)(+) transhydrogenase	*pntAA*	nadph regeneration	0,539
Q9I6G0	L-threonine dehydratase	*ilvA1*	amino-acid biosynthesis	0,539
Q9HTF1	Low specificity L-threonine aldolase	*ltaE*	biosynthetic process	0,538
Q9HVA0	Acetolactate synthase	*ilvI*	biosynthetic process	0,538
Q9I140	aminomethyltransferase	*gcvT2*	catabolic process	0,538
Q9I2T8	Periplasmic chaperone PpiD	*ppiD*	isomerase (mol)	0,538
Q9I2U7	Cysteine--tRNA ligase	*cysS*	protein biosynthesis	0,537
Q9HYT1	Solute-binding protein family 3/N-terminal domain-containing protein		transmembrane transport	0,537
G3XD24	Methyl-accepting chemotaxis protein PctA	*pctA*	chemotaxis	0,534
Q9HWC4	Transcription termination/antitermination protein NusG	*nusG*	dna-templated transcription termination	0,534
Q9I4W0	Phosphoribosylaminoimidazole-succinocarboxamide synthase	*purC*	purine biosynthesis	0,534
Q9I6H6	DUF4399 domain-containing protein		unknown	0,534
Q9HYD5	NAD-dependent malic enzyme	*maeA*	metabolic process	0,534

P43903	Quinone oxidoreductase	*qor*	zinc ion binding	0,533
Q9I3L9	Thiosulfate-binding protein	*cysP*	transport	0,533
Q9I526	Cysteine synthase B	*cysM*	stringent response	0,533
Q9HWE2	Large ribosomal subunit protein uL16	*rplP*	translation	0,532
Q9HWF5	Protein translocase subunit SecY	*secY*	protein transport	0,532
Q9HXY1	Methionine aminopeptidase	*map*	proteolysis	0,531
Q9HTT8	HemY N-terminal domain-containing protein		metabolic process	0,530
Q9HX20	Gamma-glutamyl phosphate reductase	*proA*	amino-acid biosynthesis	0,530
Q9I2S4	Probable enoyl-CoA hydratase/isomerase		fatty acid metabolism	0,528
Q9I2U1	ATP-dependent Clp protease proteolytic subunit 1	*clpP1*	cellular response to antibiotic	0,528
Q9I1M2	2-oxoisovalerate dehydrogenase subunit alpha	*bkdA1*	catabolic process	0,526
Q9HX44	Probable acyl-CoA dehydrogenase		oxidoreductase (mol)	0,526
Q9I083	Probable outer membrane protein			0,526
Q9I0A4	Phenylalanine--tRNA ligase beta subunit	*pheT*	protein biosynthesis	0,526
Q9HVZ7	UDP-N-acetylmuramoyl-tripeptide--D-alanyl-D-alanine ligase	*murF*	cell wall organitation	0,525
Q9I1Z6	Alcohol dehydrogenase (Zn-dependent)		oxidoreductase (mol)	0,525
Q9HXG8	ABC transporter substrate-binding protein			0,525
Q9I0A3	Phenylalanine--tRNA ligase alpha subunit	*pheS*	protein biosynthesis	0,524
Q9HVU9	PmbA protein	*pmbA*	proteolysis	0,523
Q9HW15	Probable short-chain dehydrogenase	*speA*	fatty acid elongation	0,523
Q9HW02	UDP-N-acetylmuramate--L-alanine ligase	*murC*	cell wall organitation	0,522
Q9LCT3	Protein translocase subunit SecA	*secA*	protein transport	0,521
Q9HTJ1	NAD/NADP-dependent betaine aldehyde dehydrogenase	*betB*	aldehyde dehydrogenase	0,520

Q9HU15	Beta-ketoacyl-[acyl-carrier-protein] synthase FabY	*fabY*	biosynthetic process	0,520
Q9XCL6	Glutamate--tRNA ligase	*gltX*	protein biosynthesis	0,520
Q9I067	FAD dependent oxidoreductase domain-containing protein		oxidoreductase (mol)	0,519
Q9I0J4	NADH-quinone oxidoreductase subunit I	*nuoI*	aerobic respiration	0,519
P72173	Aspartate aminotransferase	*aspC*	biosynthetic process	0,518
Q9HXK5	2-isopropylmalate synthase	*leuA*	amino-acid biosynthesis	0,518
Q9I5A4	Acetate kinase	*ackA*	metabolic process	0,517
Q9HUV9	Bifunctional purine biosynthesis protein PurH	*purH*	biosynthetic process	0,516
Q9I2U0	ATP-dependent Clp protease ATP-binding subunit ClpX	*clpX*	catabolic process	0,516
Q9I5U3	Chaperone SurA	*surA*	protein folding	0,515
Q9HUE4	Homocysteine synthase	*metY*	biosynthetic process	0,514
Q9HZP5	Electron transfer flavoprotein-ubiquinone oxidoreductase		electron transport	0,514
P38103	4-hydroxy-tetrahydrodipicolinate reductase	*dapB*	biosynthetic process	0,513
Q9HWE5	Large ribosomal subunit protein uL14	*rplN*	translation	0,513
Q9HY11	AMP nucleosidase			0,513
Q9I612	Probable acyl-CoA dehydrogenase		oxidoreductase (mol)	0,512
Q9I6J1	Putrescine-binding periplasmic protein SpuD	*spuD*	transport	0,512
Q9ZFK4	2-dehydro-3-deoxyphosphooctonate aldolase	*kdsA*	biosynthesis	0,512
G3XD76	Tetrahydropicolinate succinylase	*dapD*	biosynthetic process	0,511
P24474	Nitrite reductase	*nirS*	electron transport	0,510
Q9I1N7	mannose-1-phosphate guanylyltransferase	*pslB*	biofilm formation	0,510
Q9I6A7	DUF4426 domain-containing protein		unknown	0,510
Q9I7B7	Glycine--tRNA ligase alpha subunit	*glyQ*	protein biosynthesis	0,510

Q9I0J8	NADH-quinone oxidoreductase subunit E	*nuoE*	aerobic respiration	0,509
Q9I777	DUF1993 domain-containing protein		unknown	0,508
Q9HY84	Argininosuccinate synthase	*argG*	amino-acid biosynthesis	0,508
O69077	Aspartokinase	*lysC*	biosynthetic process	0,507
P72158	N5-carboxyaminoimidazole ribonucleotide synthase	*purK*	biosynthetic process	0,507
Q9HUV8	Phosphoribosylamine--glycine ligase	*purD*	biosynthetic process	0,507
P50987	Argininosuccinate lyase	*argH*	biosynthetic process	0,506
Q51344	Aspartate-semialdehyde dehydrogenase	*asd*	biosynthetic process	0,506
Q9HU38	AsmA domain-containing protein		regulation of protein targeting to membrane	0,506
Q9HZJ2	Fatty acid oxidation complex subunit alpha	*fadB*	fatty acid metabolism	0,506
Q9HU43	imidazole-4-carboxamide isomerase	*hisA*	biosynthetic process	0,505
Q9HUA5	Glucans biosynthesis protein G	*opgG*	metabolic process	0,505
Q9HTQ2	Alanine racemase, catabolic	*dadX*	peptidoglycan biosynthetic process	0,504
Q9I2Y7	Phospho-2-dehydro-3-deoxyheptonate aldolase		amino-acid biosynthesis	0,504
Q51422	Aspartate--tRNA(Asp/Asn) ligase	*aspS*	atp binding	0,503
Q9I003	ATP-dependent RNA helicase DeaD	*deaD*	catabolic process	0,503
Q9HXJ5	Histidine--tRNA ligase	*hisS*	protein biosynthesis	0,502
Q9HYV7	Beta-ketodecanoyl-[acyl-carrier-protein] synthase		biosynthetic process	0,502
Q9HUK5	Phosphoserine phosphatase		biosynthetic process	0,501
G3XD72	Probable purine-binding chemotaxis protein		chemotaxis	0,500
Q9HVF1	Probable malate:quinone oxidoreductase 2	*mqo2*	oxidoreductase activity	0,499
Q51342	Amidophosphoribosyltransferase	*purF*	biosynthetic process	0,498
Q9I3I5	Cell division protein ZipA	*zipA*	cell cycle-cell division	0,498

Q9HY12	MBL fold metallo-hydrolase		snrna processing	0,498
Q9HZC5	Aminopeptidase N	*pepN*	proteolysis	0,498
Q9I271	Cysteine protease		proteolysis	0,496
Q9I2Q7	Sulfite reductase	*cysI*	iron binding (mol)	0,496
Q9HXI0	Sec translocon accessory complex subunit YajC		protein transport	0,496
O52761	Large ribosomal subunit protein bL17	*rplQ*	translation	0,495
Q9I6J0	Spermidine-binding periplasmic protein SpuE	*spuE*	transport-virulence	0,495
P77915	oxidase	*hemN*	biosynthetic process	0,494
Q9HW72	Pyruvate kinase	*pykA*	glycolitic process	0,494
P47203	Cell division protein FtsA	*ftsA*	cell division	0,493
Q9I2U8	Glutamine--tRNA ligase	*glnS*	protein biosynthesis	0,493
Q9HYZ6	Septum site-determining protein MinD	*minD*	cell division	0,493
P11436	Aliphatic amidase	*amiE*	catabolic process	0,492
G3XD20	Probable periplasmic serine endoprotease DegP-like	*mucD*	protein quality control	0,491
Q9I4S5	Pyridoxine/pyridoxamine 5'-phosphate oxidase	*pdxH*	pyridoxine biosynthesis	0,490
P48247	Glutamate-1-semialdehyde 2,1-aminomutase	*hemL*	biosynthetic process	0,489
Q9HT19	ATP synthase gamma chain	*atpG*	atp synthesis	0,489
Q9HVU0	Cell shape-determining protein MreB	*mreB*	regulation of cell shape	0,489
Q9I5Q9	N-acetyl-gamma-glutamyl-phosphate reductase	*argC*	amino-acid biosynthesis	0,489
Q9I6J2	Putrescine--pyruvate aminotransferase	*spuC*	biosynthetic process	0,489
Q9HXI8	Cysteine desulfurase IscS	*iscS*	ion binding (mol)	0,489
Q59641	Peptidyl-prolyl cis-trans isomerase A	*ppiA*	protein folding	0,488
P00106	Cytochrome c4	*cc4*	electron transport	0,487

Q9I7C4	Beta sliding clamp	*dnaN*	dna replication	0,486
Q9HX33	Leucine--tRNA ligase	*leuS*	protein biosynthesis	0,486
Q9HXE5	ATP-dependent RNA helicase RhlB	*rhlB*	catabolic process	0,486
P38100	Carbamoyl phosphate synthase large chain	*carB*	biosynthetic process	0,485
Q9HU83	Urocanate hydratase	*hutU*	catabolic process	0,485
Q9I2T9	Lon protease	*lon*	cellular response to antibiotic	0,485
Q9HZZ0	L,D-TPase catalytic domain-containing protein		cell shape	0,485
Q9HUM6	Adenylosuccinate synthetase	*purA*	biosynthetic process	0,484
Q9I0T2	Probable acyl-CoA dehydrogenase		catabolic process	0,483
Q9X6R0	Polyamine aminopropyltransferase 1	*speE1*	biosynthesis	0,482
Q9HX46	AMP nucleosidase	*amn*	metabolic process	0,482
Q9HYU4	Long-chain-fatty-acid--CoA ligase	*fadD1*	catabolic process	0,482
Q9I558	Acetyl-coenzyme A synthetase 1	*acsA1*	biosynthetic process	0,481
Q9HU53	2,3-bisphosphoglycerate-independent phosphoglycerate mutase	*gpmI*	metabolic process	0,480
Q9HZ63	Ubiquinone biosynthesis O-methyltransferase	*ubiG*	biosynthetic process	0,480
Q9HVU7	Metalloprotease TldD		proteolysis	0,479
Q9I3G6	Probable ferredoxin		electron transport	0,479
Q9I5I5	3-hydroxyisobutyryl-CoA hydrolase		catabolic process	0,479
P47205	UDP-3-O-acyl-N-acetylglucosamine deacetylase	*lpxC*	biosynthetic process	0,478
Q9I6H4	FAD-binding PCMH-type domain-containing protein		biosynthetic process	0,478
Q9HVC2	Ribosome-binding ATPase Ych	*ychF*	ribosomal large subunit binding	0,477
P50601	Tol-Pal system protein TolB	*tolB*	cell division/ protein import	0,476
Q9HU41	Imidazoleglycerol-phosphate dehydratase	*hisB*	biosynthetic process	0,476

Q9I2R2	Probable oxidoreductase		oxidoreductase (mol)	0,476
Q9I576	4-hydroxyphenylpyruvate dioxygenase	*hpd*	catabolism	0,476
Q9HVL8	GTPase Obg	*obg*	ribosome biogenesis	0,475
P57112	Soluble pyridine nucleotide transhydrogenase	*sthA*	metabolic process	0,474
Q9I1F5	Probable glyceraldehyde-3-phosphate dehydrogenase		catabolic process	0,474
Q9I7C2	DNA gyrase subunit B	*gyrB*	dna replication	0,474
Q9I0L2	tRNA-specific 2-thiouridylase MnmA	*mnmA*	trna processing	0,474
G3XD74	serine-type D-Ala-D-Ala carboxypeptidase	*dacC*	proteolysis	0,473
Q9HUM7	Ribonuclease R	*rnr*	catabolic process	0,473
Q9I3D8	Uncharacterized protein PA1579		lipid binding (mol)	0,472
Q9HT06	Membrane protein insertase YidC	*yidC*	protein transport	0,471
Q9HTX5	Aminomethyltransferase	*gcvT*	transaminase activity	0,471
P14165	Citrate synthase	*gltA*	tricarboxylic acid cycle	0,470
Q9I553	Alanine--tRNA ligase	*alaS*	protein biosynthesis	0,470
Q9I5Y8	Transketolase	*tktA*	transferase (mol)	0,469
Q51485	Porin B	*oprB*	porin activity	0,468
Q9HU50	Probable carboxyl-terminal protease		protein secretion	0,468
Q9HVW8	ATP phosphoribosyltransferase	*hisG*	biosynthetic process	0,468
Q9I1M0	Lipoamide acyltransferase component of branched-chain alpha-keto acid dehydrogenase complex	*bkdB*	glycolysis	0,466
Q9I344	Chorismate synthase	*aroC*	amino-acid biosynthesis	0,466
Q9I5U2	LPS-assembly protein LptD	*lptD*	biosynthetic process	0,466
Q9HZP8	Enoyl-[acyl-carrier-protein] reductase [NADH]	*fabV*	fatty acid biosynthesis	0,466
P26276	Phosphomannomutase	*algC*	biosynthetic process	0,465

Q9HUQ6	Probable short-chain dehydrogenase		oxidoreductase activity	0,464
Q51390	Glycerol kinase 2	*glpK2*	biosynthetic process	0,462
Q9HT76	Vitamin B12-dependent ribonucleotide reductase	*nrdJa*	biosynthetic process	0,462
Q9HW04	Arginine biosynthesis bifunctional protein ArgJ	*argJ*	biosynthetic process	0,462
Q9I456	Probable outer membrane protein			0,461
Q9I298	3-methylglutaconyl-CoA hydratase	*liuC*	catabolic process	0,460
Q9I6J9	Agmatine deiminase	*aguA*	biosynthesis	0,460
P0DP44	Polyphosphate kinase	*ppk*	biosynthetic process	0,459
Q9I5F9	Lon protease	*lon*	cellular response to antibiotic	0,459
Q9HUF1	Peptide methionine sulfoxide reductase MsrA	*msrA*	cellular response	0,458
Q9HZ15	Probable transcriptional regulator		transcription regulation	0,458
Q9HVT2	Alpha-2-macroglobulin homolog		endopeptidase inhibitor activity	0,457
Q9HX32	LPS-assembly lipoprotein LptE	*lptE*	transport	0,456
P72170	Dihydroorotase	*pyrC*	biosynthetic process	0,455
Q9HY01	S-(hydroxymethyl)glutathione dehydrogenase	*adhC*	catabolic process	0,455
O68822	Cytosol aminopeptidase	*pepA*	rna metabolic process	0,454
Q9HUG0	Probable carbamoyl transferase		biosynthetic process	0,452
Q9HV50	Phosphoglucosamine mutase	*glmM*	biosynthetic process	0,452
Q9HWD7	Large ribosomal subunit protein uL23	*rplW*	translation	0,452
Q9I513	Phosphoribosylformylglycinamidine cyclo-ligase	*purM*	purine biosynthesis	0,452
Q9I7A7	LysM domain-containing protein			0,452
Q9HZL1	Na(+)-translocating NADH-quinone reductase subunit F	*nqrF*	ion transport	0,451
Q9I0J6	NADH-quinone oxidoreductase subunit G	*nuoG*	aerobic respiration	0,451

Q9HU18	C4-dicarboxylate-binding periplasmic protein DctP	*dctP*	transmembrane transport	0,450
P52477	Multidrug resistance protein MexA	*mexA*	response to antibiotic/ transmembrane transport	0,449
Q9HVJ1	Energy-dependent translational throttle protein EttA	*ettA*	translation	0,449
O33877	3-hydroxydecanoyl-[acyl-carrier-protein] dehydratase	*fabA*	fatty acid biosynthetic process	0,448
Q9HTV1	Transcription termination factor Rho	*rho*	rna binding	0,448
Q9I4L6	Outer-membrane lipoprotein YfiB	*yfiB*		0,448
Q9I2X2	Inactive transglutaminase fused to 7 transmembrane helices			0,446
O50177	Succinylglutamate desuccinylase	*astE*	catabolic process	0,445
P28810	Methylmalonate-semialdehyde dehydrogenase	*mmsA*	catabolic process	0,445
O86428	Branched-chain-amino-acid aminotransferase	*ilvE*	biosynthetic process	0,444
Q9I028	Acyl-coenzyme A dehydrogenase		fatty acid biosynthesis	0,443
P26275	Positive alginate biosynthesis regulatory	*algR*	dna binding	0,442
Q9HVY9	BON domain-containing protein		response to osmotic stress	0,442
Q9I2A2	fumarylacetoacetase	*fahA*	catabolism	0,442
Q9I3F6	Methyl-accepting chemotaxis protein Aer	*aer*	chemotaxis	0,442
Q9I747	Protein hcp1	*hcp1*	protein binding (mol)	0,441
Q9HV48	ATP-dependent zinc metalloprotease FtsH	*ftsH*	cell division	0,440
Q9HW49	UspA domain-containing protein			0,440
Q9I6E0	Dihydroxy-acid dehydratase	*ilvD*	amino-acid biosynthesis	0,440
G3XCU6	Transcriptional regulator CysB	*cysB*	regulation of dna-templated transcription	0,439
P48372	DNA gyrase subunit A	*gyrA*	dna binding	0,438
Q9HXI1	Protein translocase subunit SecD	*secD*	protein transport	0,436

Q9HU67	Large ribosomal subunit assembly factor BipA	*typA*	biofilm formation, response to antibiotic	0,434
Q9I407	Glutaminase-asparaginase	*ansB*	catabolic process	0,434
Q9I1F8	Probable oxidoreductase		oxidoreductase (mol)	0,431
P52002	Multidrug resistance protein MexB	*mexB*	response to antibiotic/ transmembrane transport	0,430
Q9HXZ2	Acetyl-coenzyme A carboxylase carboxyl transferase subunit alpha	*accA*	lipid metabolism	0,430
Q9HZF8	Dihydroorotate dehydrogenase (quinone)	*pyrD*	pyrimidine biosynthesis	0,429
Q9HYI7	Leucine dehydrogenase	*ldh*	metabolic process	0,428
Q9HXZ4	CTP synthase	*pyrG*	biosynthesis	0,427
Q9HTV8	Membrane fusion protein biotin-lipoyl		no function	0,426
Q9I658	DUF2780 domain-containing protein		unknown	0,426
Q9HW87	Formyltetrahydrofolate deformylase	*purU1*	biosynthetic process	0,425
Q9HVB9	Carbonic anhydrase		cellular response	0,424
O50175	N-succinylarginine dihydrolase	*astB*	catabolic process	0,423
P52111	Glycerol-3-phosphate dehydrogenase	*glpD*	metabolic process	0,422
Q9HXP9	Small ribosomal subunit protein bS16	*rpsP*	translation	0,422
Q9I0I4	Methyl-accepting chemotaxis protein TlpQ	*tlpQ*	chemotaxis	0,420
G3XCT6	Chemotaxis protein CheA		chemotaxis	0,419
Q9HUW8	DUF2333 family protein			0,419
O33407	Esterase EstA	*estA*	cell motility	0,416
Q9HZJ3	3-ketoacyl-CoA thiolase	*fadA*	fatty acid metabolism	0,416
Q9HTY8	S1 motif domain-containing protein		translation	0,415
Q9HUJ8	DNA topoisomerase 4 subunit B	*parE*	chromosome segregation	0,414
P28811	3-hydroxyisobutyrate dehydrogenase	*mmsB*	catabolic process	0,412

P72157	N5-carboxyaminoimidazole ribonucleotide mutase	*purE*	biosynthetic process	0,411
Q9HXJ7	Outer membrane protein assembly factor BamB	*bamB*	protein transport	0,410
Q9I1L9	Dihydrolipoyl dehydrogenase	*lpdV*	oxidoreductase (mol)	0,405
Q9HYL2	Nitrous-oxide reductase	*nosZ*	oxidoreductase (mol)	0,405
Q9I3G5	Cbb3-type cytochrome c oxidase subunit	*ccoP1*	electron transport	0,404
Q9HWS8	Probable dehydrogenase		biosynthetic process	0,403
Q9I1I5	Glucose dehydrogenase	*gcd*	oxidoreductase (mol)	0,401
Q9HXB0	Peptide chain release factor 3	*prfC*	protein biosynthesis	0,400
Q9I0T0	Probable short-chain dehydrogenase		metabolic processes	0,393
Q9I2A9	3-oxoadipate CoA-transferase	*dhcB*	catabolic process	0,391
Q9I5G8	Elongation factor 4	*lepA*	protein biosynthesis	0,388
Q9I4T3	Probable outer membrane protein		monoatomic ion transport	0,387
Q9HVW7	UDP-N-acetylglucosamine 1-carboxyvinyltransferase	*murA*	cell wall organization	0,379
Q9HY79	Bacterioferritin	*bfrB*	ion transport	0,378
Q9HXI2	Protein translocase subunit SecF	*secF*	protein transport	0,377
O82852	Uridylate kinase	*pyrH*	biosynthetic process	0,370
Q9HVW0	Arabinose 5-phosphate isomerase KdsD	*kdsD*	biosynthetic process	0,357
Q9HWF8	Small ribosomal subunit protein uS11	*rpsK*	translation	0,353
Q9HXY4	Outer membrane protein assembly factor BamA	*opr86*	protein insertion into membrane	0,353
Q9HX05;Q9I2C4	Probable aldehyde dehydrogenase		oxidoreductase (mol)	0,345
G3XCX1	nitrate reductase (quinone)	*narG*	nitrate metabolic process	0,343
Q9I689	ATP-dependent RNA helicase RhlE	*rhlE*	ribosome biogenesis	0,330
Q9HWE8	Small ribosomal subunit protein uS14	*rpsN*	translation	0,321

P72174	UvrABC system protein B	*uvrB*	dna binding	0,275
Q9X4G0	Homogentisate 1,2-dioxygenase	*hmgA*	catabolism	0,267
Q9I702	Malonate-semialdehyde dehydrogenase	*bauC*	biosynthetic process	0,266
Q9HZ67	Bifunctional chorismate mutase/prephenate dehydratase	*pheA*	amino-acid biosynthesis	0,266
Q9I406	Glutathione hydrolase proenzyme	*ggt*	glutathione biosynthesis	0,252
Q9HVM9	Type 4 fimbrial biogenesis protein PilX	*pilX*	pilus assembly	0,250
Q9HTL5	NAD-dependent epimerase/dehydratase domain-containing protein		aldehyde dehydrogenase	0,237
Q9HYZ3	Probable M18 family aminopeptidase 2	*apeB*	proteolysis	0,230
Q51480	Protein NirF	*nirF*	biosynthesis of heme d1 of nitrite reductase	0,228
Q9HVK8	endopeptidase La		proteolysis	0,227
Q9I524	GTP pyrophosphokinase	*relA*	stringent response	0,215
G3XDA2	3-oxoacyl-[acyl-carrier-protein] synthase 2	*fabF1*	fatty acid biosynthetic process	0,191
Q9HT16	ATP synthase subunit b	*atpF*	atp synthesis	0,000
Q9HT22	Bifunctional protein GlmU	*glmU*	cell wall organization	0,000
Q9HT80	DNA polymerase I	*polA*	dna binding	0,000
Q9HTN7	Probable binding protein component of ABC dipeptide transporter		dipeptide transport	0,000
Q9HUA6	Glucans biosynthesis glucosyltransferase H	*opgH*	metabolic process	0,000
Q9HV69	Pantothenate synthetase	*panC*	biosynthetic process	0,000
Q9HVV9	3-deoxy-D-manno-octulosonate 8-phosphate phosphatase KdsC		biosynthetic process	0,000
Q9HVW9	Histidinol dehydrogenase	*hisD*	biosynthetic process	0,000
Q9I297	Methylcrotonyl-CoA carboxylase, beta-subunit	*liuB*	catabolic process	0,000
Q9I2B0	3-oxoadipate CoA-transferase	*dhcA*	catabolic process	0,000
Q9I2U6	Bifunctional protein FolD	*folD*	amino-acid biosynthesis	0,000

Q9I3N3	Cytochrome c-type biogenesis protein CcmE	*ccmE*	cytochrome c-type biogenesis	0,000
Q9I5H3	Probable two-component response regulator		transcription	0,000
Q9HWZ6	Inorganic pyrophosphatase	*ppa*	ion binding (mol)	0,000
Q9HXJ8	GTPase Der	*der*	ribosome biogenesis	0,000
Q9HZA3	3-isopropylmalate dehydratase large subunit	*leuC*	amino-acid biosynthesis	0,000
Q9I0M2	Thioredoxin reductase	*trxB1*	oxidoreductase (mol)	0,000
