# Supplementary material for: Human histone fragments display antibacterial properties against Pseudomonas aeruginosa
Source: Front Immunol. 2026 Aug 12;17:1879460. doi: 10.3389/fimmu.2026.1879460 (PMC13506288; doi:10.3389/fimmu.2026.1879460)
Supplement: Supplementary file 2 [file DataSheet2.pdf]

Supplementary Figures

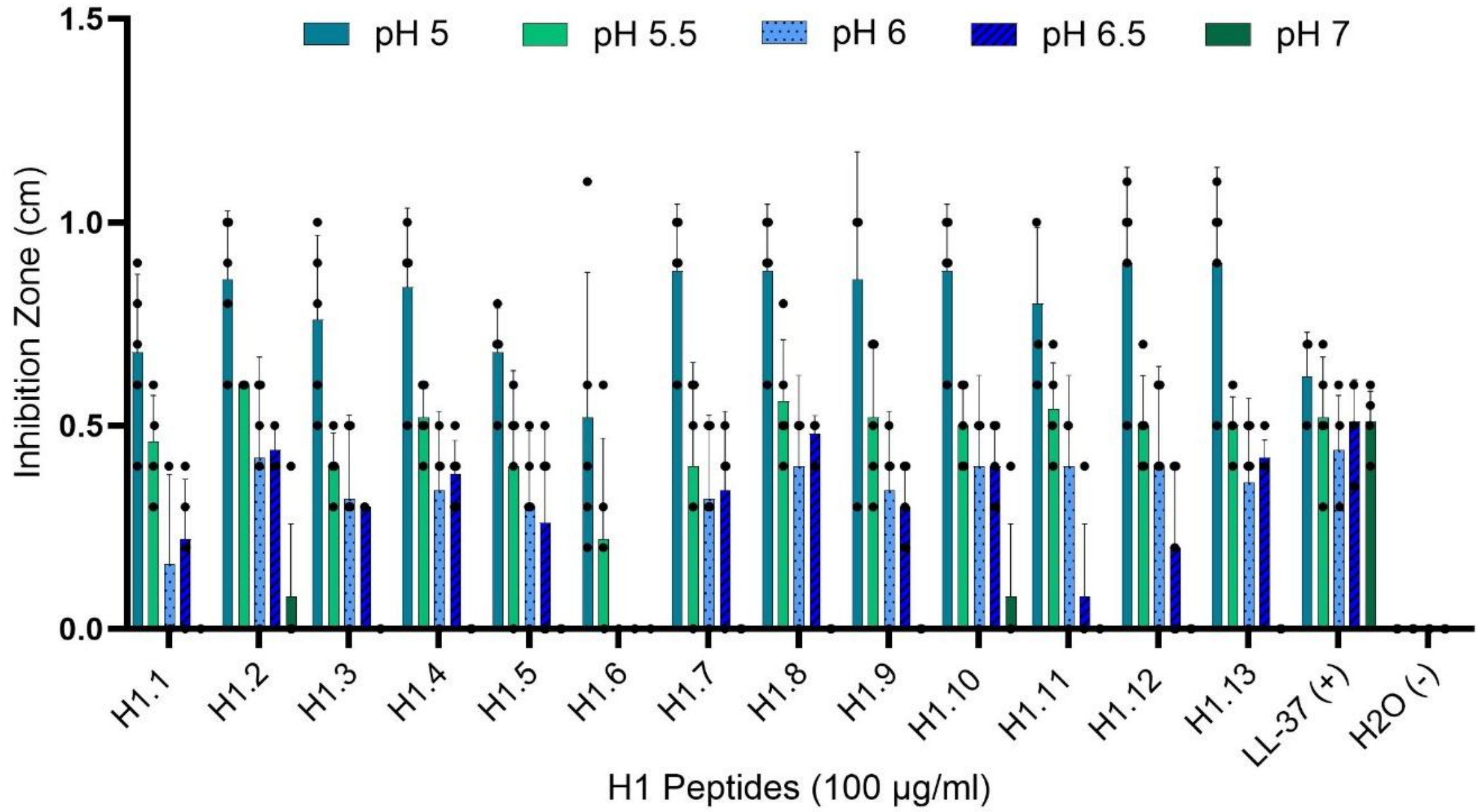

Figure S1. **H1 peptide fragments in different pH.** The 13 peptide fragments were tested against *P. aeruginosa* in different pH media in a radial diffusion assay.

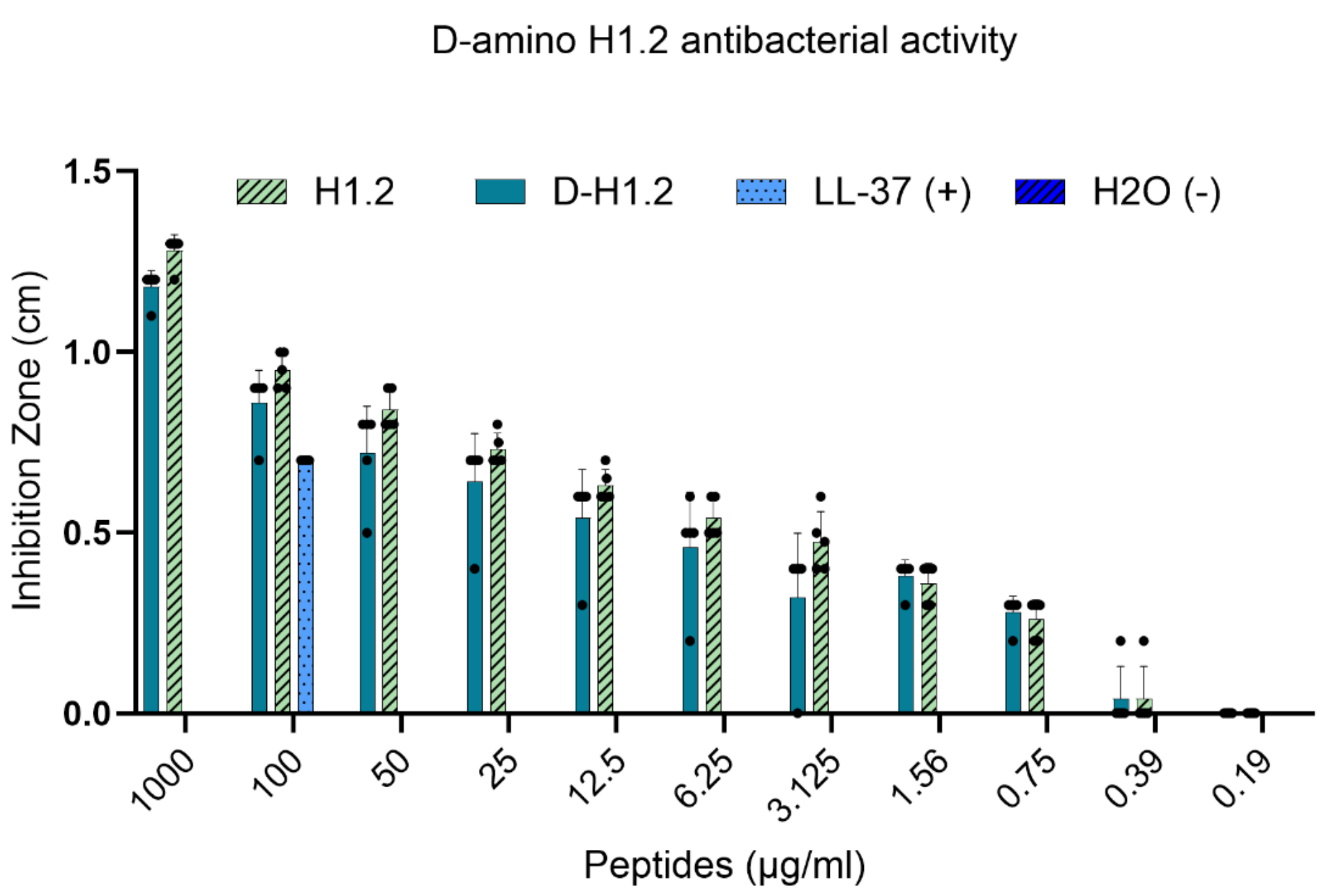

Figure S2. **H1.2 and D-amino H1.2 peptides in RDA.** The antibacterial activity tested at lower concentrations of H1.2 and D-amino H1.2 against *P. aeruginosa* in RDA assay. Both forms of the peptide the L-amino acid and the D-amino acid function similarly in inhibiting the growth of *P. aeruginosa*.

A.

B.

C.

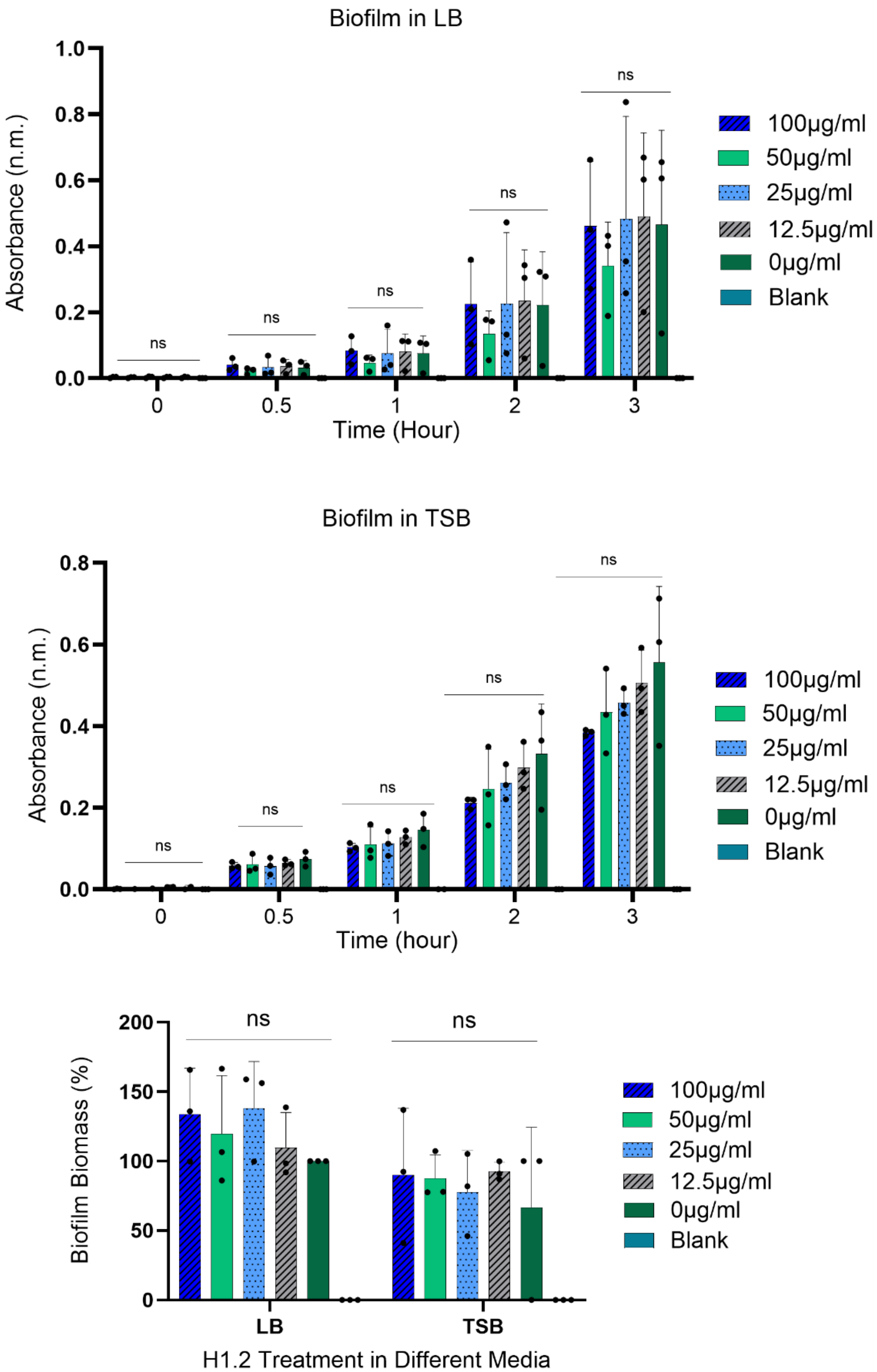

Figure S3. **H1.2 Histone peptide does not inhibit biofilm formation.** Incubating H1.2 of different concentrations with *P. aeruginosa* overnight at 37°C does not interfere with the viability of the bacteria within the formed biofilm in LB **(A)** or TSB **(B)** media. **(C).** Crystal violet assay of H1.2 tested against *P. aeruginosa* biofilm in LB and TSB media shows no significant reduction in the biofilm biomass formation.
